# Trivalent influenza vaccine adverse symptoms analysis based on MedDRA terminology using VAERS data in 2011

**DOI:** 10.1186/s13326-016-0056-2

**Published:** 2016-05-13

**Authors:** Jingcheng Du, Yi Cai, Yong Chen, Cui Tao

**Affiliations:** 1 The University of Texas School of Biomedical Informatics, 7000 Fannin St Suite 600, Houston, TX 77030 USA; 2 The University of Texas School of Public Health, 1200 Pressler Street, Houston, TX 77030 USA; 3 Department of Biostatistics and Epidemiology, University of Pennsylvania, 423 Guardian Drive, Philadelphia, PA 19104 USA

**Keywords:** Trivalent influenza virus vaccine, VAERS, MedDRA

## Abstract

**Background:**

Trivalent Influenza Virus Vaccine (FLU3) is a traditional flu vaccine to protect people against three different flu viruses, including influenza A H1N1 virus, an influenza A H3N2 virus and one B virus.

**Methods:**

We searched Vaccine Adverse Event Reporting System (VAERS) for US reports after FLU3 vaccination in the year of 2011. We conducted descriptive analyses on symptoms from serious reports (i.e., death, life-threatening illness, hospitalization, prolonged hospitalization, or permanent disability). We then further grouped these symptoms to the System Organ Classes (SOC) based on the MedDRA Terminology using NCBO Web Services. We fitted zero-truncated Poisson regression models to estimate the average number of symptoms per subject and compared it across different age groups and between genders. In addition, we compared the risk of occurrence for an SOC across different age groups and between genders by using logistic regression models. Finally, we constructed the pairwise correlation matrix of the SOCs by calculating Spearman’s rank correlation coefficients.

**Results:**

We identified 638 unique serious FLU3 reports from year 2011. There are 1410 unique symptoms from these reports. Descriptive statistics shows that the most common symptom and symptom pair are Pyrexia and Guillain-Barre syndrome – Hypoesthesia respectively. The estimated average number of symptoms per subject in the study cohort is 8.74 (95 % CI 6.76, 10.73). There are statistically significant differences in number of symptoms among four age groups and between genders. Age category and gender are significantly associated with several individual SOCs. Pairwise correlation matrix shows that “Endocrine disorders” and “Neoplasms benign, malignant and unspecified (incl cysts and polyps)” are strongly correlated.

**Conclusions:**

This paper reports a novel method that combining statistical analyses with terminology grouping using VAERS data. The analyses revealed differences of reactions among different age groups and between genders and correlation on both symptoms and System Organ Class level independently. The results may lead to additional studies to uncover factors contributing to the individual differences in susceptibility to influenza infection. This method can also be applied to other vaccine types and conduct similar analysis.

**Electronic supplementary material:**

The online version of this article (doi:10.1186/s13326-016-0056-2) contains supplementary material, which is available to authorized users.

## Background

Influenza (flu) is a contagious respiratory illness caused by influenza viruses, which may cause mild to severe illness including hospitalization or even death. Certain groups of people, such as the elders, young children, and those with certain health conditions, are at high risk for serious flu complications [1]. The primary and most used method for the control of influenza and its complications are influenza vaccines. Over the years, hundreds of millions of Americans have received seasonal influenza vaccines to protect themselves against the flu viruses. Commonly, the side effects following flu vaccinations are mild, including symptoms such as soreness, redness or swelling at the injection sites, headache, muscle aches and nausea after the shot. Serious adverse reactions, however, could happen, which may cause some life-threating illness even death.

Clinical trials are generally not large enough to detect rare influenza vaccine adverse events. In 1990, Vaccine Adverse Event Reporting System (VAERS) was created as a passive surveillance system to accept reports of adverse events following any US licensed vaccines form providers, health care workers, and the public [2]. VAERS is co-administered by Center for Disease Control and Prevention (CDC) and the Food and Drug Administration (FDA). It is one of the largest databases containing adverse events reported in temporal association with vaccination. Since 1990, VAERS has received more than 400,000 vaccine adverse event reports, which makes it one of the most important sources to detect rare vaccine adverse events. Although VAERS cannot usually prove the causal relationships between vaccines and adverse events, it could be used to detect signals to be tested with more rigorous methods [3].

For years, trivalent Influenza Virus Vaccine (FLU3) is the traditional flu vaccine to protect people against three different flu viruses, including an influenza A H1N1 virus, an influenza A H3N2 virus and one B virus [4]. Among all the VAERS reports, FLU3 is the most common vaccine type reported. Thus VAERS is a very important data source for studying FLU3 adverse events.

Much work has been done on the study of influenza adverse events using VAERS data [5–7]. Most of them only deal with some specific symptoms on the FLU3 vaccine adverse reports. A single vaccine and AE association, however, should not be considered as an isolated event. The associations of other vaccines with the same AE and other AEs with the same vaccine should also be taken into consideration [8].

Our research takes advantage of the Medical Dictionary for Regulatory Activities (MedDRA) terminology system for semantically grouping the VAERS adverse symptoms, which are already coded using MedDRA terms [2]. The MedDRA Terminology is the international medical terminology developed under the auspices of the International Conference on Harmonization (ICH) of Technical Requirements for Registration of Pharmaceuticals for Human Use. MedDRA Terminology has a five-level structural hierarchy. They are lowest Level Term (LLT), Preferred Term (PT), High Level Term (HLT), High Level Group Term (HLGT), and System Organ Class (SOC). VAERSSYMPTOMS contains symptoms terms that are in the level of LLT [9]. VAERS used symptom terms from PT, which always have its own identical term as LLT. The full MedDRA has 72,637 LLT symptoms and VAERS uses 9593 of them (13 %) [10]. System organ class (SOC) is the highest level of the hierarchy that provides the broadest concept for data retrieval, which comprises grouping by etiology, manifestation site and purpose. MedDRA has 26 different types of SOCs and each LLT is at least linked to one SOC [9].

As the number of unique symptoms is relatively large in VAERS, we consider them in the SOCs to facilitate further statistical analyses. We leveraged The National Center for Biomedical Ontology (NCBO) web service to automatically map LLTs to their corresponding SOCs. The NCBO offers a range of Web services that would allow users to access various biomedical terminologies and ontologies and to identify terms from controlled terminologies and ontologies that can describe and index the contents of online data sets (data annotation) [11]. All the MedDRA terminology system is stored in JSON format on the NCBO web services and each term is a JSON node. We used NCBO web services to search the hierarchical information of the symptom terms in the VAERS reports and assigned each symptom term one primary SOC. After grouping the symptoms to SOCs, we conducted multiple statistical analyses on the SOC level.

## Materials and methods

### Data source

We searched the VAERS for US reports after FLU3 vaccination in year 2011 and extracted serious reports (i.e., death, life-threatening illness, hospitalization, pro-longed hospitalization, or permanent disability). VAERS raw data of each year contains three Comma-separated-value (CSV) files: VAERSDATA.CSV, VAERSVAX.CSV and VAERSSYMPTOMS.CSV. VAERSDATA is about the patients’ demographic information, lab test, symptom text and outcomes. VAERSVAX is about vaccine types. VAERSSYMPTOMS is about symptoms that are equivalent to the PT TERM from the MedDRA codebook. These three tables are linked by using VAERS_ID. For each report, the VAERS also provides annotations for post-vaccination symptoms in MedDRA terms. To facilitate further statistical analyses, we further grouped these symptoms based on the MedDRA SOC using the NCBO Web Services [11].

### Descriptive analysis

We calculated descriptive statistics including the number of reports, symptoms, and unique symptoms in the selected reports. We also calculated the frequency of each symptom and co-occurrence of symptom pairs.

We grouped the reports in five age groups based on cut points (0.5, 17, 49, 64) suggested by CDC [12]. The frequency of observations in age category (0 to 0.5) is relatively small (*n* = 14) compared to other age categories. In order to be consistent with our previous analysis, we excluded those subjects in our data analysis, which lead to 1663 subjects [13].

### Grouping symptoms using MedDRA terminology

SOC is the highest level of the MedDRA terms, which comprises grouping by etiology, manifestation site or purpose. Each LLT is linked to only one PT and each PT is linked to at least one SOC. This indicates that each LLT could be grouped to more than one SOC. To avoid double counting, we will need to identify the primary SOC for each term [9]. The rules to assign SOC to the symptoms, however, are complicated, which needs expert reviews that could be time consuming and expensive.

Our study proposed a simple way to group them into SOCs by using the international agreed order of the SOC list (see Table 1). The order of the SOCs was based upon the relative importance of each SOC, which is determined by the Expert Working Group [9]. First, we retrieved all possible SOCs a VAERS symptom term belongs to. We applied Depth-first Search (DFS) algorithm by using recursive tree-traversing method to find all the SOCs that is linked by a symptom term. If one symptom term belongs to several SOCs, we choose the SOC that ranked highest as its primary SOC. If a report has N symptoms that belong to the same specific SOC, we count N times of that specific SOC. Table 2 shows the sample data set (partial) we prepared for further analysis.

**Table 1:** International Agreed Orders of SOCs

SOC	Order
Infections and infestations	1
Neoplasms benign, malignant and unspecified (incl cysts and polyps)	2
Blood and lymphatic system disorders	3
Immune system disorders	4
Endocrine disorders	5
Metabolism and nutrition disorders	6
Psychiatric disorders	7
Nervous system disorders	8
Eye disorders	9
Ear and labyrinth disorders	10
Cardiac disorders	11
Vascular disorders	12
Respiratory, thoracic and mediastinal disorders	13
Gastrointestinal disorders	14
Hepatobiliary disorders	15
Skin and subcutaneous tissue disorders	16
Musculoskeletal and connective tissue disorders	17
Renal and urinary disorders	18
Pregnancy, puerperium and perinatal conditions	19
Reproductive system and breast disorders	20
Congenital, familial and genetic disorders	21
General disorders and administration site conditions	22
Investigations	23
Injury, poisoning and procedural complications	24
Surgical and medical procedures	25
Social circumstances	26

**Table 2:** Sample data set we prepared for further analysis (partial, data of year 2011)

ID	……	SOC7	SOC8	SOC9	SOC10	……
413830		0	1	0	0	
413836		0	0	0	0	
413913		0	3	0	0	
413937		0	11	0	2	
413946		0	5	1	0	
413959		0	5	0	0	
413966		3	15	3	0	
413993		1	5	0	0	
413994		1	3	0	0	
……						……

ID refers to the report ID number. The number in a cell refers to the number of appearance in the corresponding SOC in that report

### Statistical methods

After grouping the symptoms into 26 SOCs, we explored the grouped data with regression models and correlation analysis. Specifically, we estimated the average number of symptoms per subject given stratified age groups and gender. For individual SOC, we fitted logistic regression to evaluate the association of occurrence of an SOC with age and gender. A rank-based correlation matrix is also estimated to investigate the correlation among SOCs.

#### Zero-truncated Poisson regression

As each subject has at least one SOC to be reported (i.e. the number of SOCs > 0), we fitted a zero-truncated Poisson regression model (a modified model of Poisson regression by excluding the probability mass at 0) to conduct data analysis [14]. Firstly, we fitted a model with intercept only to estimate the average number of symptoms for all subjects. After that, we included covariates of age category and gender in the model to estimate and compare the number of symptoms in different age and gender strata.

#### Logistic regression

To explore the association of the occurrence of individual SOC with age and gender, we conducted a logistic regression with covariates of age group and gender [15]. The original count number of SOC is dichotomized into binary outcomes (1: SOC ≥ 1, 0: SOC = 0).

#### Spearman rank correlation coefficient

As the number of SOCs is highly right skewed and non-normally distributed, we use a nonparametric correlation coefficient to measure the correlation structure among SOCs. This rank based correlation coefficient is known as Spearman’s ρ [16]. We constructed a correlation matrix of SOCs to present the pairwise correlation coefficients between SOCs.

## Results

**Fig. 1:**
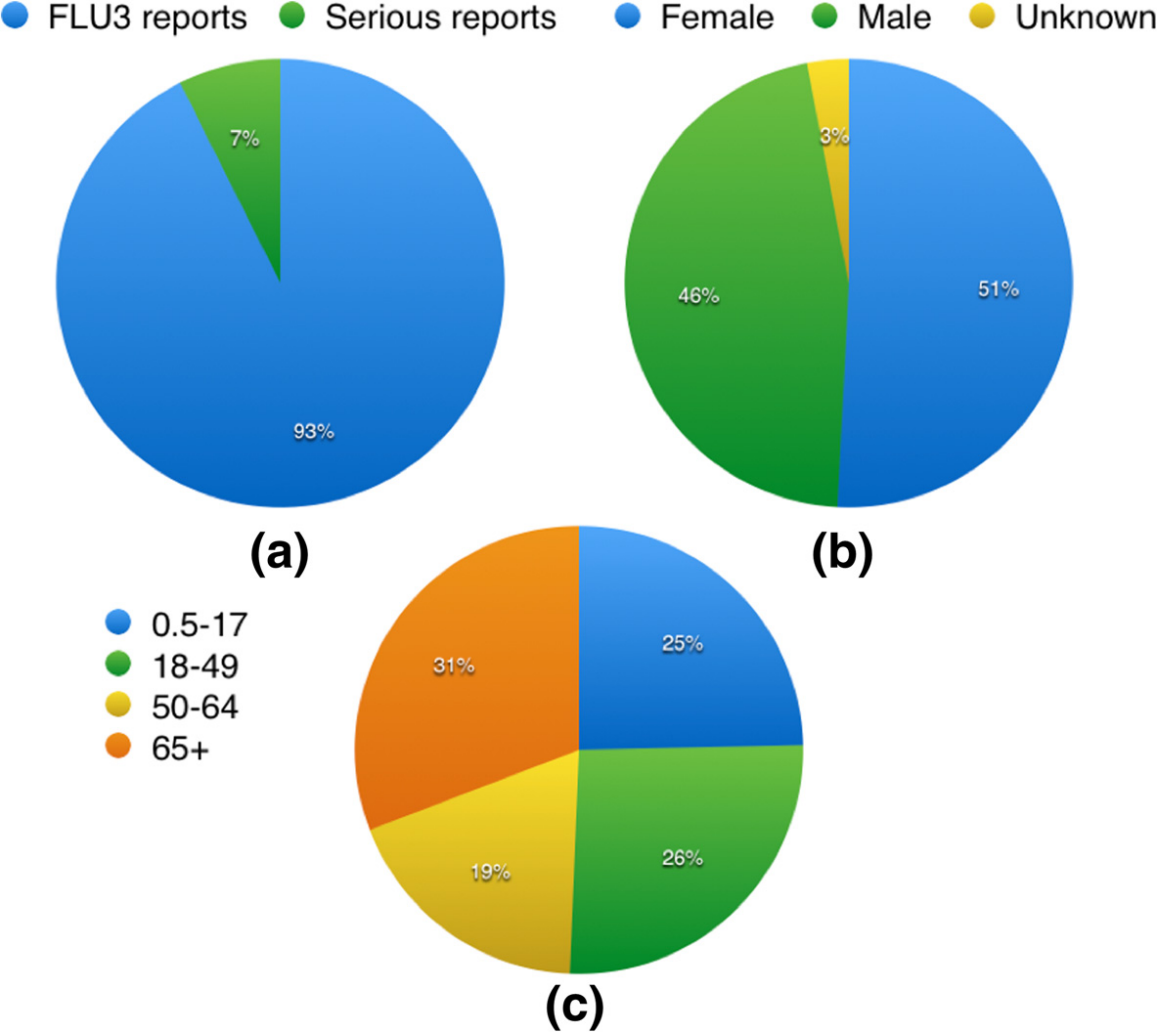
Descriptive results of 2011 VAERS FLU3 data. **a** the proportion of serious reports of the total FLU3 reports. **b** the gender distribution of these serious reports. **c** the age distribution of these serious reports

During the study period (year 2011), VAERS received 7986 FLU3 reports; 638 were serious. Out of the 638 reports, 324 were for female patients, 295 were for male patients, and 19 were for unknown sex, see Fig. 1. Out of these reports, there were 5447 symptom terms (not unique) appeared in total, which were grouped into 26 SOCs. The most frequent SOCs in the 638 reports are nervous system disorders, general disorders, and administration site condition and investigations. Please note that some symptom terms cannot be grouped to any MedDRA SOCs. There were 48 symptom terms (21 of them are unique) can’t be mapped into MedDRA SOCs, including “Drug exposure during pregnancy”, “Herpes zoster multi-dermatomal” and etc. (The complete list can be seen in Additional file 1: Table S1A). This may due to the MedDRA version differences between the NCBO Web Services and VAERS. As the number of these symptoms is relatively small (0.9 %), we did not take these symptoms into consideration.

**Fig. 2:**
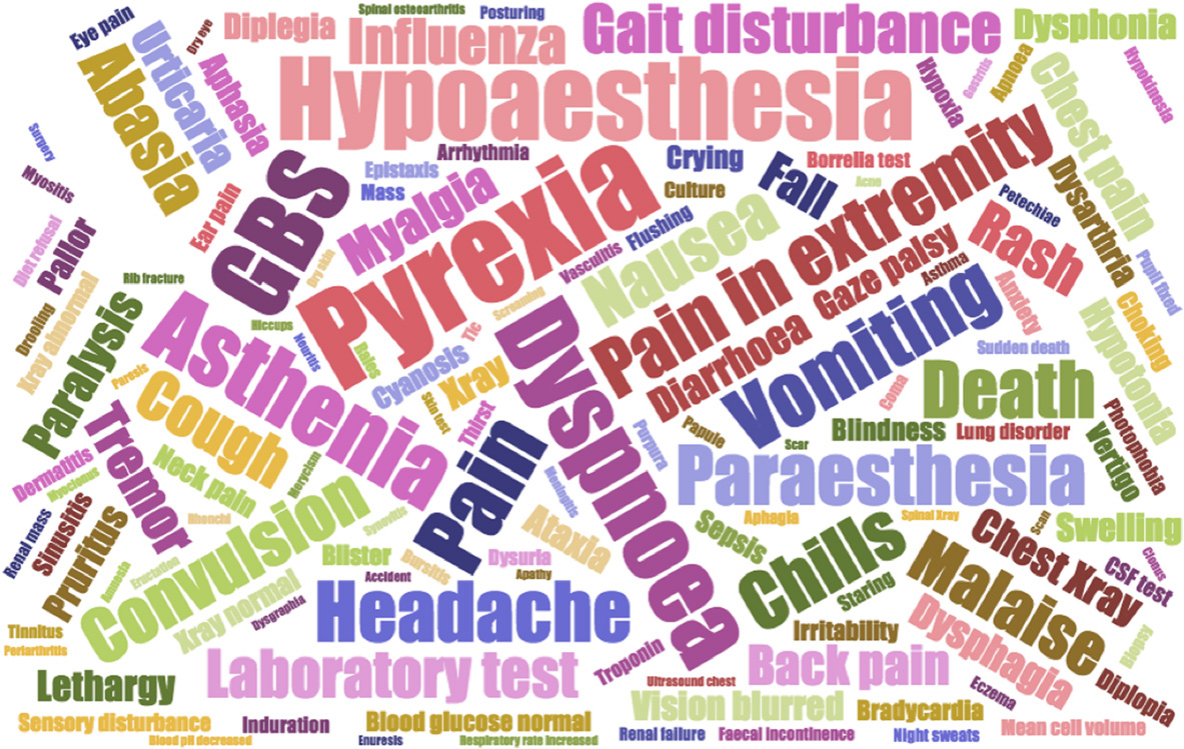
Symptoms frequency visualization for year 2011, the bigger the frequency the larger the font size of that symptom

We then calculated the frequency of each symptoms and symptom concurrences. The most frequent symptoms happened after FLU3 vaccination in year 2011 were Pyrexia (131 times), Hypoaesthesia (95 times), and Guillain-Barre syndrome (90 times), the visualization result can be seen in Fig. 2. (The complete results can be seen in Additional file 1: Table S1B). The most frequent symptom co-concurrences were Guillain-Barre syndrome + Hypoaesthesia (24 times).

Analysis using zero-truncated Poisson model with intercept only indicated that the estimated average number of symptoms per subject in the study cohort is 8.74 (95 % CI: 6.76, 10.73). The results from fitting zero-truncated Poisson regression with covariates age groups and gender suggest that there are statistically significant differences in the numbers of symptoms among four age groups and between different genders. The average number of symptoms per year for a female patient with age between 0.5 years and 17 years is estimated as 7.76 (95 % CI: 5.76–9.76). We use this group as the reference age group. The youngest age group (0.5–17 years) has the smallest number of symptoms per year, followed by age group 4 (>64 years), age group 2 (17–49) and finally age group 3 (49–64 years). The average number of symptoms for subjects of 17–49 years old is 1.13 times of the average number of symptoms for subjects of 0.5–17 years old with the same gender. This is consistent with previous reports about different immune responses after vaccination for different age groups. For example, there is high-dose influenza vaccine available for elders (>65) because ageing decreases the body's ability of immune response after vaccination [17]. In addition, the males have 3.2 % lower number of symptoms. This is also consistent with previous studies that female experience more adverse reactions to influenza vaccine [18]. We plotted the estimated residuals versus fitted values of zero-truncated Poisson regression mode. We found that the residuals are generally small and scattered around zero for most of the data points with only a few extreme values. This suggests that the zero-truncated Poisson regression model may fit the data well.

Analysis on individual SOCs also revealed some interesting results. Overall, there are 15 SOCs that show significant association with the age groups or between genders. The males have fewer responses for most SOCs except SOC1 (infections and infestations, 48.1 % more). Males are 0.369, 0.552 and 0.535 times less likely to have SOC 9 (Eye disorders), SOC 11 (Cardiac disorders) and SOC 12 (Vascular disorders) than females respectively. For SOC 14 (Gastroin-testinal disorders) and SOC 1 (Infections and infestations), female has significant higher possibilities than male. For SOC 1 (Infections and infestations), SOC 9 (Eye disorders), SOC 12 (Vascular disorders), and SOC 17 (Musculoskeletal and connective tissue disorders), people who are older than 17 show significant higher possibilities to get those adverse symptoms. Age group 3 (49–64) shows significant higher chance of experiencing symptoms relevant to infections and infestations, immune system disorders, eye disorders, vascular disorders, musculoskeletal and connective tissue disorders, surgical and medical procedures, and social circumstances compared to age group 1 (0.5–17) with the same gender. To evaluate the overall goodness of fit of the logistic regression model for each SOC, we conducted Hosmer-Lemeshow test [15] and calculated the corresponding *p*-values. Most of the p-values for all 26 tests are much smaller than 0.05, which suggests that the logistic regression model fits the data well.

**Fig. 3:**
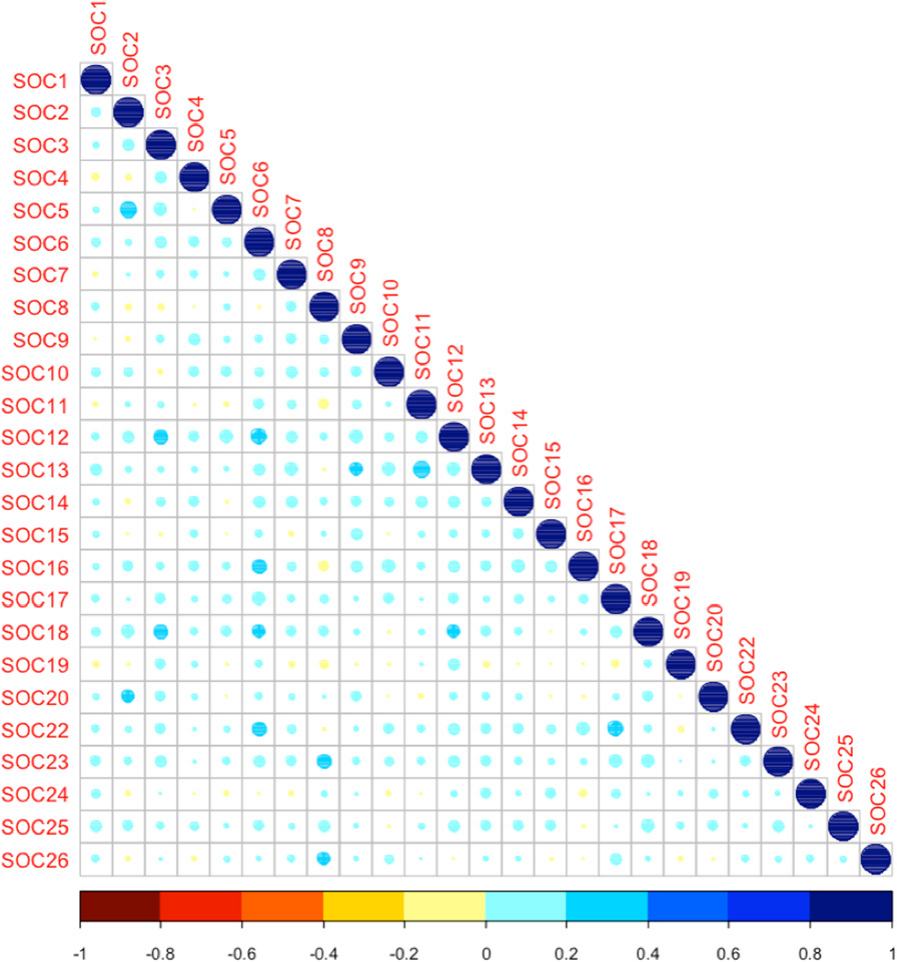
The pairwise correlation matrix of SOCs determined by Spearman’s method

We also calculated the pairwise correlation matrix of SOCs determined by Spearman’s method [16]. Fig. 3 shows the correlation plot. The color and area of spot represents the strength of correlation between SOCs. The threshold to assert a correlation used by us is whether the correlation coefficient is larger or equal than 0.2 as suggested by Evans [19]. As illustrated in Fig. 3, SOC 5 (Endocrine disorders) has the strongest correlation with SOC 2 (Neoplasms benign, malignant and unspecified (incl cysts and polyps)). Besides, we find that SOC 23 (Investigations) has a correlation with SOC 8 (Nervous system disorders). We can also find that SOC 22 (General disorders and administration site conditions) has correlations with SOC6 (Metabolism and nutrition disorders), and SOC 17 (Musculoskeletal and connective tissue disorders). In addition, SOC 12 (Vascular disorders) shows correlations with SOC 3 (Blood and lymphatic system disorders), and SOC 6 (Metabolism and nutrition disorders). In addition, SOC 18 (Renal and urinary disorders) has correlations with SOC 3 (Blood and lymphatic system disorders), SOC 6 (Metabolism and nutrition disorders) and SOC 12 (Vascular disorders).

## Conclusions and future work

This paper reports a novel method that combining statistical analyses with terminology grouping using VAERS data to study Trivalent Influenza Vaccine. Our preliminary statistical analyses reveal differences of reactions among different age groups and between genders. To our best knowledge, there are very few studies about the adverse events analysis on MedDRA SOC level. Most of our findings on the relationship between adverse events with individual difference have not been reported by other studies. The results may lead to additional studies to uncover factors contributing to the individual differences in adverse reactions to influenza vaccination.

For the limitations of this paper, due to the multiple inheritance nature of MedDRA, many symptom terms can be mapped to more than one SOCs. The order list we used to assign the primary SOC may be subjective. Ontology of Adverse Events (OAE) could provide a better hierarchy than MedDRA. The current version of OAE, however, does not include all the symptoms in the VAERS (coded in MedDRA) yet. We will consider using OAE to map the symptom terms as the OAE grows.

There are a few future directions we would like to pursue: (1) we will extend and apply the methodology to more VAERS reports over different years; and (2) this method can also be applied to other vaccine types and conduct similar analysis.

## Additional file

Additional file 1: Table S1A. Symptom terms that can’t be mapped into SOC level in the year 2011. **Table S1B.** Symptoms and their frequency for year 2011. Top 50 frequently occurred symptoms in serious FLU3 reports in the year of 2011. (DOCX 16 kb)
