# Supplementary material for: Trivalent influenza vaccine adverse symptoms analysis based on MedDRA terminology using VAERS data in 2011
Source: J Biomed Semantics. 2016 May 13;7:13. doi: 10.1186/s13326-016-0056-2 (PMC5192591; doi:10.1186/s13326-016-0056-2)
Supplement: Additional file 1: Table S1A. — Symptom terms that can’t be mapped into SOC level in the year 2011. Table S1B. Symptoms and their frequency for year 2011. Top 50 frequently occurred symptoms in serious FLU3 reports in the year of 2011. (DOCX 16 kb) [file 13326_2016_56_MOESM1_ESM.docx]

# Additional file 1

**Table S1A** Symptom terms that can’t be mapped into SOC level in the year 2011.

| Drug exposure during pregnancy |
| --- |
| Herpes zoster multi-dermatomal |
| Anaemia haemolytic autoimmune |
| Open wound |
| Idiopathic thrombocytopenic purpura |
| Gastrostomy tube insertion |
| Intra-uterine death |
| Hypoaesthesia facial |
| Antibiotic resistant Staphylococcus test negative |
| Intestinal functional disorder |
| Autoimmune thrombocytopenia |
| Skin chapped |
| Leukocytoclastic vasculitis |
| Echography normal |
| Myopericarditis |
| Nerve root lesion |
| Colonic polyp |
| Collapse of lung |
| Diagnostic procedure |
| Antibiotic resistant Staphylococcus test positive |
| Nuclear magnetic resonance imaging spinal cord abnormal |

**Table S1B** Symptoms and their frequency for year 2011. Top 50 frequently occurred symptoms in serious FLU3 reports in the year of 2011.

| **Symptoms** | **Frequency** |
| --- | --- |
| Pyrexia | 131 |
| Hypoaesthesia | 95 |
| Guillain-Barre syndrome | 90 |
| Dyspnoea | 83 |
| Asthenia | 70 |
| Muscular weakness | 70 |
| Pain | 65 |
| Vomiting | 54 |
| Headache | 51 |
| Paraesthesia | 49 |
| Pain in extremity | 47 |
| Chills | 46 |
| Death | 45 |
| Nuclear magnetic resonance imaging | 40 |
| Computerised tomogram normal | 39 |
| Convulsion | 39 |
| Nausea | 38 |
| Malaise | 37 |
| Lumbar puncture | 37 |
| White blood cell count increased | 36 |
| Abasia | 35 |
| Intensive care | 33 |
| Cough | 32 |
| Oedema peripheral | 32 |
| Gait disturbance | 32 |
| Laboratory test | 31 |
| Dizziness | 31 |
| Fatigue | 30 |
| Computerised tomogram head | 29 |
| Erythema | 28 |
| Rash | 28 |
| Influenza | 28 |
| Laboratory test normal | 27 |
| Blood test | 26 |
| Injection site pain | 25 |
| Tremor | 25 |
| Nuclear magnetic resonance imaging normal | 25 |
| Immunoglobulin therapy | 24 |
| CSF protein increased | 23 |
| Blood culture negative | 23 |
| Myalgia | 22 |
| Condition aggravated | 22 |
| Febrile convulsion | 22 |
| Computerised tomogram | 21 |
| Fall | 21 |
| Back pain | 21 |
| Endotracheal intubation | 21 |
| Decreased appetite | 20 |
| Cellulitis | 20 |
| Body temperature increased | 20 |
